# Predictors of sexual and reproductive health knowledge and utilization of services among adolescents in Ghana's Adaklu district

**DOI:** 10.4314/gmj.v58i1.9

**Published:** 2024-03

**Authors:** Clare Westerman, Margaret Gyapong, Evelyn K Ansah, Desmond Klu, Matilda Aberese-Ako, Maxwell A Dalaba

**Affiliations:** 1 Boston University Chobanian & Avedisian School of Medicine, MA, USA; 2 Institute of Health Research, University of Health and Allied Sciences, Ho, Ghana

**Keywords:** sexual health, reproductive health, adolescent health, Ghana, sexual education

## Abstract

**Objective:**

To explore factors associated with adolescents' sexual and reproductive health (SRH) knowledge and their engagement with educational and clinical services

**Design:**

Regression analysis of secondary data collected during a community survey

**Setting:**

Adaklu district, Volta Region, Ghana

**Participants:**

221 adolescent caregiver pairs

**Main outcome measures:**

The study employed three main outcome measures: (1) adolescents' level of SRH knowledge (assessed via questionnaire), (2) membership in district-sponsored adolescent health clubs (AHCs), and (3) ever-utilization of clinical SRH services.

**Results:**

Greater SRH knowledge was significantly associated with older age, AHC membership, and relying primarily on teachers or friends for SRH information. Increased odds of AHC membership were observed among females (AOR = 2.38, 95% CI 1.14-4.95); those who had communicated with one parent about sexual issues (OR 2.70, 95% CI 1.17-6.21); and those with a history of transactional sex (OR 5.53, 95% CI 1.04-29.37). Decreased odds were observed among adolescents whose caregivers were educated to the primary level (AOR = 0.24, 95% CI = 0.07-0.79). Overall, utilization of clinical SRH services was low, but higher odds were detected among individuals reporting a history of forced sex (AOR = 117.07, 95% CI 3.82-3588.52) and those who had discussed sexual issues with both of their parents (AOR = 13.11, 95% CI 1.85-92.93).

**Conclusions:**

Awareness of the predictors of knowledge, AHC involvement, and clinical service utilization can empower adolescent SRH initiatives—both present and future—to enhance their teaching, develop targeted outreach to underserved groups, and promote engagement with key clinical resources.

**Funding:**

This work has been supported by grants from the International Development Research Centre [108936] (IDRC), Canada.

## Introduction

The sexual and reproductive health (SRH) of Ghanaian adolescents is of critical concern. The compounding effects of stigma, inexperience, financial dependence, and structural inaccessibility combine with the physiological vulnerability of a still-developing body to place Ghana's young people at elevated risk of numerous complications ranging from sexual abuse to sexually transmitted infections (STI), obstetric fistula, and maternal mortality.^1^,^2^ Nationwide, approximately 37.1% of females and 21.3% of males aged 15-19 have had sexual intercourse; over a quarter of them began doing so before the age of 15.^3^ The burden of STI remains high among adolescents, and nearly one in every five young women in the nation gives birth to her first child before reaching the age of 18 years.^3^,^4^ Despite these adverse outcomes, just 17.8% of unmarried, sexually active young people are using a modern method of contraception.^5^ This problem persists despite rising geographic access to healthcare facilities and the presence of a nationwide mandate that schools provide “comprehensive sexuality education” (CSE) as part of both the primary- and secondary-school curriculum.^6^,^7^

Past studies assessing SRH awareness among Ghanaian adolescents have reported wide variations in the extent of knowledge possessed,^8^–^11^, and few have examined the factors that contribute to the observed trends. In addition, aside from the national CSE curriculum, educational initiatives have largely been short-term or limited in geographic scope, lacking the government support required for widespread scale-up and sustainability.^12^-^14^ More research is needed to determine how adolescents obtain accurate SRH information and to identify the subpopulations most in need of additional education.

In 2015, the Volta region's Adaklu district gained national attention for its 23.2% rate of adolescent pregnancy—one of the highest in the nation.^15^,^16^ The following year, district authorities established the adolescent health club (AHC) initiative, which sought to implement a network of community-based youth groups dedicated to increasing health knowledge and reducing SRH-related morbidity and mortality. Led by a trained health worker, these clubs host monthly meetings to provide adolescents with information about key wellness topics such as personal hygiene, menstruation, family planning, and navigating social and sexual relationships.^17^,^18^ Although preliminary reports suggest a promising impact on behavior,^18^ systematic explorations of the demographic profile of those served—and not yet served—have been limited thus far. Examining the factors associated with AHC membership offers a valuable opportunity to enhance the reach and impact of the initiative in a community greatly in need of additional support.

Lastly, although essential, education is just one component of effective SRH care, for adolescents cannot act upon their knowledge without access to the appropriate resources. Despite the presence of 15 public clinics and health centers across Adaklu district, just 10% of adolescent residents have ever obtained clinical SRH support or counseling.^19^,^20^ This low utilization of services presents a substantial cause for concern, particularly against the backdrop of the epidemic of adolescent pregnancy. Nonetheless, the factors predicting young people's engagement with the available services remain largely unexamined. Likewise, although past research has demonstrated a link between effective SRH education and care-seeking behavior,^21^ the association between AHC involvement and service utilization in Adaklu has not yet been assessed.

**Figure 1:**
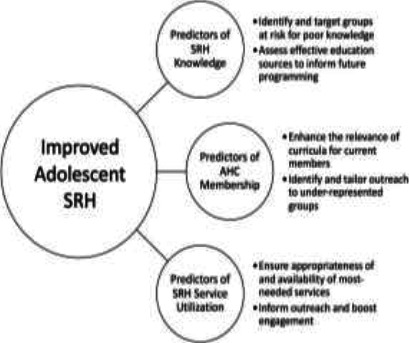
Logic model illustrating the three key variables of study and their proposed utility in improving adolescent health

To address the aforementioned knowledge gaps, the present study sought to explore the social and demographic predictors of (1) SRH knowledge, (2) AHC membership, and (3) SRH service utilization among adolescents in the Adaklu district. The results of this study can provide valuable insights to both present and future SRH programs, empowering them both to better serve their current clientele and to develop targeted initiatives to engage adolescents not yet benefitting from the interventions (See Figure 1).

## Methods

### Study Population and Setting

This secondary analysis utilized data collected during a community survey exploring the SRH knowledge, attitudes, and behaviors of young people aged 10-19 and their caregivers in the Adaklu district of Ghana's Volta region. Adaklu district is a rural, low-income, largely agrarian community of 38,649 individuals, 23.5% of which are adolescents.^16^ Approximately 84 AHCs are currently active, providing services to over 1,800 local youth.^18^

### Data Analysis

#### Predictor and outcome variables

Analyses targeted three main outcome variables: (1) level of SRH knowledge (2) self-reported membership in an AHC, and (3) ever-use of SRH services provided by a health worker or clinic. Predictor variables included a variety of demographic, social, and sexual factors. Complete lists of the predictor variables included in each analysis can be found in Table 2.

**Table 2:** Results of multivariate regression analyses (Reference categories for each predictor variable are listed first)

	Model 1: predictors of SRH knowledge score	Model 2: predictors of AHC membership	Model 3: predictors of SRH service utilization
**Regression Type**	Linear	Logistic	Logistic
**Measure of Association**	Coefficient (p-value)	AOR (95% CI)	AOR (95% CI)
**Predictor Variables**			
**Sex**			
**Male**			
**Female**	−0.57 (0.128)	2.38 (1.14-4.95)*	0.042 (0.07-2.62)
**Age group**			
**10-14**			
**15-19**	2.14 (0.001***)	1.47 (0.64-3.38)	2.06 (0.45-36.77)
**Educational attainment**			
**No formal schooling**			
**Primary**	−0.83 (0.102)	1.96 (0.72-5.33)	0.06 (0.00-1.14)
**Junior secondary or higher**	0.23 (0.686)	2.78 (0.90-8.58)	0.40 (0.03-5.52)
**Current school attendance**			
**Not attending**			
**Attending**	−0.07 (0.904)	2.45 (0.75-7.96)	0.33 (0.04-2.37)
**Caregiver's education level**			
**No formal schooling**			
**Primary**	0.29 (0.566)	0.24 (0.07-0.79)*	2.02 (0.24-16.64)
**Junior secondary or higher**	0.85 (0.122)	0.54 (0.18-1.61)	0.95 (0.10-8.67)
**Living situation**			
**Living with no parents**			
**Living with one parent**	0.59 (0.324)	1.40 (0.55-3.56)	0.81 (0.11-6.13)
**Living with both parents**	−0.48 (0.210)	1.19 (0.46-3.10)	1.05 (0.11-9.94)
**Sub-district**			
**Waya**			
**Helekpe**	−0.06 (0.907)	5.67 (1.82-17.63)**	0.23 (0.02-2.30)
**Wumenu**	−0.90 (0.068)	2.80 (1.07-7.28)*	1.11 (0.19-6.31)
**Sofa**	0.10 (0.864)	1.04 (0.33-3.22)	0.12 (0.01-1.42)
**Ahunda**	−0.53 (0.335)	5.18 (1.78-15.09)**	1 (empty)
**Relationship history**			
**No history of romantic relationships**			
**Past or current romantic partner**	0.83 (0.066)	0.72 (0.29-1.79)	2.72 (0.40-18.51)
**Sexual history**			
**Never had sexual intercourse**			
**Ever had sexual intercourse**	−0.68 (0.243)	1.17 (0.34-4.00)	7.09 (0.96-52.43)
**History of transactional sex**			
**Never participated**			
**Participated**	−0.86 (0.207)	5.53 (1.04-29.37)*	0.27 (0.02-3.49)
**History of forced sex**			
**Never experienced**			
**Experienced**	−0.53 (0.525)	0.62 (0.11-3.52)	117.07 (3.82-3588.52)**
**History of unwanted touching**			
**Never experienced**			
**Experienced**	79 (0.111)	1.29 (0.46-3.59)	0.81 (0.12-5.38)
**AHC membership**			
**Nonmember**			
**Member**	1.44 (0.001***)		4.09 (0.54-31.15)
**SRH service utilization**			
**Never utilized**			
**Utilized**	1.23 (0.057)	1.95 (0.49-7.74)	-
**Communication with parents about sexual issues**			
**No parents**			
**Only one parent**	0.63 (0.120)	2.70 (1.17-6.21)*	1.21 (0.15-9.56)
**Both parents**	0.49 (0.396)	1.92 (0.59-6.28)	13.11 (1.85-92.93)*
**Caregiver's opinion on adolescent dating^b^**			
**Disapprove**			
**Approve**	0.21 (0.791)	0.50 (0.10-2.64)	0.19 (0.01-3.51)
**Main source of information about sex and reproduction**			
**Other/None**			
**School teacher**	2.59 (0.022*)		
**Family**	1.28 (0.288)	-	-
**Friends**	3.07 (0.010**)	-	-
**Health workers/AHC**	1.76 (0.041)	-	-
**Media**	2.23 (0.154)	-	-

AOR = adjusted odds ratio

CI = confidence interval

* significant at α ≤ 0.05

** significant at α ≤ 0.01

*** significant at α ≤ 0.001

- = not included in model

#### Measuring knowledge levels

The adolescent version of the original survey included several questions designed to assess respondents' basic knowledge of SRH. Individual answers to these questions were used to calculate a “SRH knowledge score,” ranging from Univariate Analysis 0 to 16, for each participant.

All correct answers were weighted such that each question represented a total of one point, and the distribution was characterized with summary statistics and histogram analysis. Sample questions and additional detail about the tabulation process can be found in Appendix 1.

### Multivariate analysis

Multivariate analyses were undertaken to ascertain factors associated with the three primary outcomes of interest: (1) SRH knowledge, (2) AHC membership, and (3) utilization of SRH services. Linear regression was used to identify predictors of enhanced SRH knowledge, while binary logistic regressions were used to explore trends in AHC membership and service utilization. All variables in each regression were entered in a single step, and p ≤ 0.05 was adopted as a general threshold for significance. All analyses were performed in Stata/SE 17.0.

### Ethical considerations

Ethical approval for the baseline survey and subsequent analyses was provided by the Research Ethics Committee of the University of Health and Allied Sciences, Ghana (reference number UHAS-REC A.8 [3] 18-19). Informed consent was obtained from all participants prior to their completion of the survey. To further protect respondents' confidentiality in the current study, all included data were transmitted and analyzed in a de-identified manner.

## Results

### Sociodemographic characteristics of participants

A summary of participant characteristics is presented in Table 1. In total, the sample consisted of 221 adolescents ranging in age from 10 to 19 years, with 45.25% in the 10-14 age group and 54.75% aged 15 to 19. More females (59.73%) than males (40.27%) were represented in the survey, and the overwhelming majority of respondents (99.10%) self-identified as Christian. Although most participants (90.05%) were actively attending school, their levels of education varied, with roughly similar percentages having completed primary (43.44%) and secondary (42.08%) school and 14.48% yet to complete their primary education. Caregiver education levels spanned a wide range, although most (57.47%) had completed at least junior secondary school. Slightly over 28% of care-givers had completed only primary school, and the remaining 14.05% had received no formal education.

**Table 1:** Sociodemographic and sexual characteristics of adolescent respondents to the sexual and reproductive health baseline community survey, 2019 (n = 221)

Characteristic	n (%)
**Sex**	
Male	89 (40.27%)
Female	132 (59.73%)
**Age group**	
10-14	100 (45.25%)
15-19	121 (54.75%)
**Religion**	
Christian	219 (99.10%)
Atheist	2 (0.90%)
**Educational attainment**	
No formal schooling	32 (14.48%)
Primary	96 (43.44%)
Junior secondary or higher	93 (42.08%)
**Current school attendance**	
Attending	199 (90.05%)
Not attending	22 (9.95%)
**Living situation**	
Living with both parents	86 (38.91%)
Living with one parent	92 (41.63%)
Living with no parents	43 (19.46%)
**Sub-district**	
Waya	43 (19.46%)
Helekpe	45 (20.36%)
Wumenu	60 (27.15%)
Sofa	30 (13.57%)
Ahunda	43 (19.46%)
**Relationship history**	
Past or current romantic partner	76 (34.39%)
No history of romantic relationships	145 (65.61%)
**Sexual history**	
Ever had sexual intercourse	42 (19.81%)
Never had sexual intercourse	170 (80.19%)
**History of transactional sex**	
Ever participated in transactional sex	22 (9.95%)
Never participated in transactional sex	199 (90.05%)
**History of sexual assault** ^**a**^	
Experienced forced sex	16 (7.24%)
Experienced unwanted touching	45 (20.36%)
None	171 (77.38%)
**Adolescent health club membership**	
Member	144 (65.16%)
Not a member	77 (34.84%)
**SRH service utilization**	
Ever utilized	22 (9.95%)
Never utilized	199 (90.05%)
**Communication with parents about sexual issues**	
Both parents	25 (11.31%)
Only one parent	61 (27.60%)
No parents	135 (61.09%)
**Main source of information about sex and reproduction**	
School teacher	93 (42.08%)
Family	34 (15.38%)
Friends	46 (20.81%)
Health workers/adolescent health club	38 (17.19%)
Media (books, magazines, films)	5 (2.26%)
Other/None	5 (2.26%)
**Caregiver's education level**	31 (14.03%)
No formal schooling	63 (28.51%)
Primary	127 (57.47%)
**Caregiver's opinion on adolescent dating** ^**b**^	
Approve	13 (5.88%)
Disapprove	208 (94.12%)

a Percents do not sum to 100% due to possibility of selecting multiple answers

b As assessed by the question “Do you approve for your adolescent girl/boy to have a boy/girlfriend at her/his present age?”

The majority of the adolescent respondents lived with either one (41.63%) or both (38.91%) biological parents, although 19.46% shared a household with neither of their parents. Between 10% and 20% of participants hailed from each of the five sub-districts.

### Sexual attitudes and behaviors

Respondents varied widely with regard to their sexual histories (see Table 1). In total, 34.39% reported a current or past romantic partner, and 19.81% had experienced sexual intercourse. Participation in transactional sex (as a payer or provider) was reported by 9.95% of the sample, and 7.24% of respondents disclosed having been subjected to forced sex. Unwanted touching of a sexual nature was a somewhat more common experience, reported by 20.36% of the youth.

Although the majority (65.16%) of participants were AHC members, less than one in ten (9.95%) had ever utilized clinical SRH services. Likewise, most of the participants (61.09%) had never discussed sexual issues with either of their parents. Instead, most accessed information about sex and sexuality primarily through their teachers (42.08%), their friends (20.81%), or community health workers (17.19%). Family members of any relation represented the primary source of sex-related information for just 15.38% of those surveyed. An overwhelming majority (94.12%) of caregivers did not believe that it was appropriate for their adolescents to be involved in romantic relationships at their current age.

### Adolescents' knowledge of SRH

**Figure 2:**
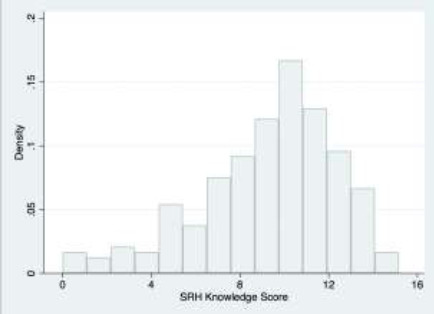
Distribution of sexual and reproductive health knowledge scores among Adaklu district adolescents, 2019. Possible scores range from 0 to 16

Figure 2 displays the distribution of SRH knowledge scores among the surveyed adolescents. Scores ranged from a minimum of 0 to a maximum of 15.17—just slightly below a perfect score of 16 points. The median score of 9.75 (IQR 7.53-11.47) represents a success rate of 61% when answering the knowledge-related questions.

Table 2, Model 1 displays the factors associated with adolescents' knowledge of SRH, as determined via linear regression (R^2^adj = 0.4268). The results of corresponding bivariate analyses for each variable, as well as those in Models 2 and 3, are collectively presented in Appendix 2. Although several factors—most notably dating history and utilization of clinical services—bordered on statistical significance, older age (p < 0.001), main source of SRH information (p = 0.002 for “school teacher,” p = 0.010 for “friends”), and membership in an AHC (p < 0.001) emerged as the only significant predictors of knowledge score. All three of these variables were associated with enhanced knowledge in comparison to their respective reference categories.

Of the three, the effects of information source were most pronounced: as compared to those who selected “other/none” as their primary source of SRH information, adolescents who had learned primarily from their friends and school teachers scored an average of 3.07 and 2.59 points higher, respectively. Of further note, AHC members scored significantly higher on average than peers both in the bivariate regression (coeff = 1.53 points, p < 0.001, see Appendix 2) and in the adjusted model (1.44, p ≤ 0.001, see Table 2).

### Predictors of adolescent Health Club membership

The significant predictors of AHC membership included several demographic and social variables (see Table 2, Model 2). Firstly, females were substantially more likely than males to participate in the clubs (AOR = 2.38, 95% CI 1.14-4.95), although age and schooling did not strongly affect the odds of club membership. In contrast, the observed relationship between caregiver education level and AHC involvement was complex. While adolescents whose caregivers had completed secondary education showed no significant difference in AHC participation as compared to peers whose caregivers had received no formal education, caregiver education to the primary level corresponded to lowered odds of club membership (OR 0.24, 95% CI = 0.07-0.79). An association was also found between AHC involvement and having discussed sexual issues with one parent (OR 2.70, 95% CI 1.17-6.21). With regard to location, the Helekpe (OR 5.67, 95% CI 1.82-17.63), Wumenu (OR 2.80, 95% CI 1.07-7.28), and Ahunda (OR 5.18, 95% CI 1.78-15.09) subdistricts exhibited significantly higher rates of AHC membership. Lastly, youth reporting a history of transactional sex were approximately five times more likely to be current members of an AHC than those who had never engaged in the practice (OR 5.53, 95% CI 1.04-29.37).

### Predictors of SRH service utilization

A logistic regression was undertaken to explore the interaction between various factors and ever-utilization of clinical SRH services. The results of this analysis are depicted in Table 2, Model 3. Most strikingly, adolescents who had experienced forced sex were over 100 times more likely than their peers to have accessed SRH services (AOR = 117.07, 95% CI 3.82-3588.52). Although the effect was less profound, individuals who communicated with both of their parents about sexual issues also had higher odds of utilization of SRH services (AOR = 13.11, 95% CI 1.85-92.93).

Notably, neither romantic history, AHC membership, nor a history of sexual intercourse significantly increased an individual's odds of having accessed services. Lastly, although the results were not fully conclusive, the complete lack of service utilization observed among respondents in the Ahunda sub-district suggests that location may play some role in shaping usage patterns as well.

## Discussion

This study utilized multivariable regression analysis of community survey data to identify the factors associated with adolescents' SRH knowledge, participation in AHCs, and ever-utilization of clinical SRH counseling or care in the Adaklu district of Ghana. These findings can inform the trajectory of future programs, empowering them to better engage young people in Adaklu and beyond.

### SRH knowledge levels

Broadly speaking, SRH knowledge among the study population was relatively low, with 50% of adolescents correctly answering fewer than two-thirds of the presented questions. This finding aligns with the results of studies performed in the Upper East, Central, and Northern regions of Ghana, which also reported low levels of SRH knowledge among adolescents in the respective regions.^8^,^22^,^23^ Notably, a survey by Amankwaa and colleagues indicated that adolescents in Kumasi, Ghana's second-largest city, possessed satisfactory knowledge of SRH; this discrepancy suggests a potential cultural or rural-urban divide in knowledge of sexual health.^24^

As evidenced by the results of the linear regression, age was a strong and significant predictor of knowledge score. This finding is not surprising, for increased age imparts adolescents with additional educational and life experiences that may enhance their knowledge of SRH. Nonetheless, at least 10.8% of young women and 6.8% of young men in Ghana initiate sexual activity before the age of 15,^5^ and a lack of information during this vulnerable period places them at heightened risk of exploitation or engagement in unsafe practices.^25^ Hence, future programs should seek to provide younger adolescents with the same breadth of SRH information available to their older peers, empowering them with the knowledge that they need *before* their lack of experience places them at risk.

The relationship between SRH knowledge level and primary information source raises several interesting points. While the results of this study further support the importance of teachers as health educators for Ghanaian students,^26^ the relatively lower knowledge scores of adolescents whose families serve as their primary sex educators—particularly compared to those who obtain their information primarily from friends—runs counter to popular theories about which sources of information are and are not “reliable.”^27^ Interestingly, while Ghanaian adolescents have expressed their desire for greater communication with their caregivers about sexual issues,^28^ surveys of Ghanaian parents have shown that many hesitate to serve as SRH educators for their children because they feel that they lack the requisite knowledge themselves.^28^,^29^ Developing parental education programs could help to address this concern by empowering care-givers with the knowledge and skills to supplement adolescents' school- and club-based learning with evidence-based information informed by family values and preferences. All the same, given lingering hesitancy on the part of some parents to engage in SRH discussions—particularly regarding traditionally-controversial topics such as contraception—it will be important to maintain the availability and quality of alternative educational sources, such as AHCs.

Lastly, the positive association between knowledge score and AHC involvement reflects favorably on the program, suggesting that the clubs may be making progress toward their goal of equipping the young people of the Adaklu district with appropriate knowledge of SRH. This finding builds upon an established body of evidence supporting the value of club-based sexual education^13^,^30^ Indeed, as of 2019, the adolescent pregnancy rate in Adaklu had decreased to approximately 12.2%, with particularly low rates among AHC members.^18^,^31^ It will be important to continue monitoring the impact of AHCs as their expansion continues.

### Adolescent Health Club participation

The data indicate that AHC membership is more common among females than among their male counterparts. This finding may reflect the particular onus often placed on girls to maintain sexual health and chastity.^32^ However, rates of male engagement in risky sexual behaviors remain high in Ghana, and young men ultimately play an equally important role in promoting safe sexual practices.^33^ Thus, AHCs should consider developing recruitment efforts tailored toward the male demographic to ensure that all adolescents have access to vital information. Additionally, given the elevated odds of club membership among adolescents who had discussed sexual issues with a parent, involving the parents of young men in these recruitment efforts may prove beneficial by reducing stigma or encouraging interest in SRH. In this effort and beyond, the clubs in Helekpe, Wumenu, and Ahunda can provide valuable models or even mentorship for AHCs in less-engaged areas as they seek to boost participation.

Lastly, the observation of higher odds of AHC membership for individuals with a history of transactional sex invites additional exploration. The association of these two practices has not been widely reported in the literature thus far. Future research into this relationship could aid AHCs in better supporting adolescents with this unique history.

### SRH service utilization

Although SRH service utilization was not found to be specifically associated with higher knowledge in the present study, the services provided at these facilities—such as contraceptive counseling, STI testing, and one-on-one consultation with qualified providers—are vital components of a comprehensive effort to promote SRH. Unfortunately, few variables emerged as significant predictors of SRH service utilization in the study context.

Indeed, neither a history of sexual intercourse nor a history of romantic relationships made adolescents more likely to have used these services. This observation is concerning, for it indicates that the vast majority of individuals most directly in need of contraceptive counseling, STI testing, and the like may not be receiving these critical services. Further qualitative research to gather the opinions of sexually-active adolescents could help to elucidate the social and structural forces discouraging them from seeking care. This need is particularly pressing in the Ahunda sub-district, where none of the 43 adolescents who participated in the survey had ever accessed clinical SRH support.

In Adaklu, adolescents who had discussed sexual issues with both of their parents (as opposed to neither) had over 13 times the odds of having accessed services, even when controlling for sexual history. This association between service utilization and adolescent-parent communication aligns with similar reports from Ethiopia.^34^ Given the prominence of perceived parental disapproval as a barrier to care-seeking among Ghanaian adolescents,^35^,^36^ communication with family members may help to alleviate these concerns, thus freeing young people to take action to protect their sexual health. Alternatively, discussing SRH with parents may enhance adolescents' awareness of the existence or value of clinical services. Whatever the explanation, the underlying message is promising: encouraging caregivers to engage their adolescents in open conversations about sexuality—particularly if coupled by enhanced parental education—can empower young people to seek out the services that promote their reproductive well-being.

Lastly, the emergence of a history of forced sex as a predictor of utilization represents a sobering but possible strength for the district, suggesting that at least some proportion of young survivors are seeking medical care after an assault. All the same, while similar to national statistics and those reported in neighboring countries,^37^,^38^ the 7.24% prevalence of rape and 20.36% prevalence of unwanted sexual touching among a group of individuals who have not even reached the age of 20 vehemently warrant a response. Proactive steps must be taken at both the structural and social levels to prevent future violations and to ensure that survivors are provided with comprehensive, appropriate, and trauma-informed care. Likewise, health workers and clinics should be aware of the potential for sexual trauma among adolescent patients when planning resource-procurement, seeking continuing education, and providing services.

### Limitations and future directions

This study was limited by the reliance on pre-collected data collected via survey. For instance, due to the sensitive nature of sexuality, the survey results are susceptible to response bias, as some participants may have felt uncomfortable disclosing stigmatized behaviors. Furthermore, as is broadly the case with cross-sectional research, the survey from which data were drawn lacked a measure of relative temporality, thus limiting this analysis' capacity to demonstrate causality in the observed relationships.

Thus, although AHC membership was significantly associated with higher SRH knowledge scores, it is difficult to say with certainty that AHC involvement was directly responsible for this trend. As the AHC initiative evolves, experimental or quasi-experimental studies—perhaps utilizing a larger sample size distributed across a broader area—should be performed to further monitor its impact.

### Summary of recommendations

Our findings have important implications for all those seeking to improve the health of Adaklu's adolescents. For AHCs themselves, the results suggest a need to improve the comprehensiveness of educational programming for younger individuals (age 10-14) to equip them to make healthy and informed choices throughout their adolescence. In further service of this goal, the current low levels of participation by males suggest an opportunity to better target outreach efforts—or, as needed, to develop supplementary programs to serve young males in the community. Concurrently, understanding the profile of adolescents already engaging with the AHCs or with clinical SRH services can aid in enhancing existing programming. Educational efforts might be expanded, for instance, to further explore key topics such as transactional sex, sexual assault prevention, and the importance of consent.

For health facilities, the general lack of uptake by adolescents demonstrates a need for broad and diverse outreach strategies to boost utilization of these crucial services across the population. Performing further research to explore local adolescents' motivations for pursuing or not pursuing services could offer valuable insights toward this end. Concurrently, the elevated odds of service utilization by adolescents who had discussed sexual issues with a parent implies that motivated caregivers might serve as useful partners in these efforts to raise awareness, dispel stigma, and remove any barriers identified. Concurrent with these outreach programs, clinical facilities must equip themselves to serve their current and future clientele by ensuring the presence of high-quality care for all adolescents, with special emphasis on ensuring the presence of comprehensive and trauma-informed services for young survivors of sexual assault.

## Conclusion

The results of this analysis reveal that the extent of SRH knowledge possessed by adolescents in Ghana's Adaklu district is significantly associated with older age, obtaining SRH information primarily from school teachers or friends, and participation in the community-based AHC initiative. In turn, AHC membership is more common among females, youth reporting a history of transactional sex, those who had discussed sexual issues with a parent, and those from the sub-districts of Helekpe, Wumenu, and Ahunda, while it is less common among individuals whose caregivers were educated to the primary level. Overall, adolescent utilization of SRH services is low, although higher odds of use were found among survivors of forced sex as well as adolescents who had discussed sexual issues with both of their parents.

Ultimately, the findings and recommendations can inform the design of future interventions to empower adolescents with the knowledge, skills, and resources to protect their sexual health and enhance their lifelong success.

## Appendix

**Appendix 1:** Formula used to calculate sexual and reproductive health knowledge scores

Question	Points Awarded
A woman can get pregnant on the very first time that she has sexual intercourse.	1 point for correct answer (Yes)
A woman stops growing after she has had sexual intercourse for the first time.	1 point for correct answer (No)
A woman is most likely to get pregnant if she has sexual intercourse halfway between her periods.	1 point for correct answer (Yes)
Which contraceptive methods have you heard of?	0.2 points for each known (spontaneous or prompted): - Pill - Injection - Condom - Emergency contraceptive pills - Withdrawal
Do you know any place or person where young people could obtain this method?	0.25 points for each yes: - Pill - Injection - Condom - Emergency contraceptive pills
There are other methods of contraception that I have not mentioned. What other methods have you heard of?	0.167 points for each additional method spontaneously listed: - IUD - Implant - Jelly/foam - Female sterilization - Male sterilization - Periodic abstinence
Have you heard of HIV or AIDS?	1 point for yes
It is possible to cure AIDS	1 point for correct answer (No)
A person with HIV always looks emaciated or unhealthy in some way	1 point for correct answer (No)
People can take a simple test to find out whether they have HIV	1 point for correct answer (Yes)
What are the signs and symptoms of a sexually transmitted disease in a man?	0.333 points for each: - Discharge from penis - Pain during urination - Ulcers/sores in genital area
And what are the signs or symptoms when a woman is infected?	0.333 points for each: - Vaginal discharge - Pain during urination - Ulcers/sores in genital area
Condoms are an effective method of preventing pregnancy	1 point for correct answer (Yes)
A condom can be used more than once	1 point for correct answer (No)
Condoms are an effective way of protecting against HIV/AIDS	1 point for correct answer (Yes)
Condoms are an effective way of protecting against sexually transmitted diseases	1 point for correct answer (Yes)

**Appendix 2:** Results of bivariate regression analyses. Reference categories for each predictor variable are listed first

	Model 1: predictors of SRH knowledge score	Model 2: predictors of AHC membership	Model 3: predictors of SRH service utilization
**Regression Type**	Linear	Logistic	Logistic
			
**Measure of Association**	Coefficient (p-value)	OR (95% CI)	OR (95% CI)
**Predictor Variables**			
**Sex**			
**Male**			
**Female**	−0.49 (0.240)	2.28 (1.29-4.01)**	2.48 (0.88-7.00)
**Age group**			
**10-14**			
**15-19**	3.05 (0.001***)	1.19 (0.68-2.07)	4.19 (1.37-12.84)*
**Educational attainment**			
**No formal schooling**			
**Primary**	−0.92 (0.114)	2.83 (0.89-4.51)	0.42 (0.01-1.99)
**Junior secondary or higher**	1.56 (0.008**)	2.21 (0.97-5.01)	1.86 (0.50-6.90)
**Current school attendance**			
**Not attending**			
**Attending**	−0.95 (0.170)	2.47 (1.01-6.02)*	0.23 (0.08-0.68)
**Caregiver's education level**			
**No formal schooling**			
**Primary**	0.36 (0.598)	0.49 (0.19-1.28)	1.13 (0.32-3.99)
**Junior secondary or higher**	−0.18 (0.767)	0.68 (0.28-1.65)	0.51 (0.15-1.80)
**Living situation**			
**Living with no parents**			
**Living with one parent**	0.09 (0.150)	1.35 (0.64-2.86)	1.19 (0.35-4.03)
**Living with both parents**	−0.73 (0.202)	1.22 (0.57-2.60)	1 (0.28-3.52)
**Sub-district**			
**Waya**			
**Helekpe**	−0.24 (0.717)	2.95 (1.19-7.30)*	0.50 (0.14-1.85)
**Wumenu**	−1.11 (0.069)	1.77 (0.80-3.94)	1.03 (0.36-2.96)
**Sofa**	−0.69 (0.339)	1.09 (0.43-2.78)	0.18 (0.02-1.52)
**Ahunda**	−0.49 (0.454)	3.15 (1.25-7.95)*	1 (empty)
**Relationship history**			
**No history of romantic relationships**			
**Past or current romantic partner**	2.07 (0.001***)	1.04 (0.58-1.87)	8.07 (2.84-22.88)***
**Sexual history**			
**Never had sexual intercourse**			
**Ever had sexual intercourse**	1.73 (0.001**)	1.44 (0.69-3.00)	11.64 (4.32-31.39)***
**History of transactional sex**			
**Never participated**			
**Participated**	0.27 (0.700)	1.48 (0.55-3.95)	4.29 (1.47-12.48)**
**History of forced sex**			
**Never experienced**			
**Experienced**	−0.50 (0.535)	1.19 (0.40-3.56)	9.85 (3.22-30.16)***
**History of unwanted touching**			
**Never experienced**			
**Experienced**	1.40 (0.006**)	1.41 (0.69-2.87)	4.85 (1.95-12.10)**
**AHC membership**			
**Nonmember**			
**Member**	1.53 (0.001***)	-	1.48 (0.55-3.95)
**SRH service utilization**			
**Never utilized**			
**Utilized**	2.11 (0.002**)	1.48 (0.55-3.95)	-
**Communication with parents about sexual issues**			
**No parents**			
**Only one parent**	0.78 (0.098)	2.38 (1.20-4.74)*	2.06 (0.71-5.99)
**Both parents**	1.11 (0.095)	1.82 (0.71-4.66)	6.17 (2.00-19.08)**
**Caregiver's opinion on adolescent dating^b^**			
**Disapprove**			
**Approve**	1.02 (0.244)	0.605 (0.20-1.87)	4.69 (1.31-16.76)*
**Main source of information about sex and reproduction**			
**Other/None**			
**School teacher**	4.97 (0.001***)	0.38 (0.04-3.52)	0.33 (0.03-3.32)
**Family**	4.37 (0.002**)	0.69 (0.07-7.07)	0.86 (0.08-9.10)
**Friends**	5.44 (0.001***)	0.39 (0.04-3.76)	0.49 (0.05-5.27)
**Health workers/AHC**	4.79 (0.001**)	1.11 (0.11-11.49)	0.34 (0.03-4.13)
**Media**	7.12 (0.001***)	1 (omitted)	1 (omitted)

OR = odds ratio

CI = confidence interval

* significant at α ≤ 0.05

** significant at α ≤ 0.01

*** significant at α ≤ 0.001

- = not included in model
